# Newborn Screening and Molecular Profile of Congenital Hypothyroidism in a Chinese Population

**DOI:** 10.3389/fgene.2018.00509

**Published:** 2018-10-29

**Authors:** Bin Yu, Wei Long, Yuqi Yang, Ying Wang, Lihua Jiang, Zhengmao Cai, Huaiyan Wang

**Affiliations:** Changzhou Maternity and Child Health Care Hospital Affiliated to Nanjing Medical University, Changzhou, China

**Keywords:** newborn screening, congenital hypothyroidism, thyroid-stimulating hormone, molecular diagnosis, gene mutation

## Abstract

To review the characteristics of newborn screening of congenital hypothyroidism (CH), we reviewed the newborn screening data, including the levels of blood spot thyroid-stimulating hormone (TSH), and serum TSH and free thyroxine (FT4), of all newborn infants who accepted the newborn screening program during the last 14 years. In total, 437,342 newborn infants underwent CH screening and 192 infants were diagnosed with CH and the incidence of CH was 1:2278. The positive rate of the initial screening was 0.96%, and the positive predictive value was 4.8%. We also designed a target sequencing panel including 13 causative genes: *DUOX2*, *TG*, *TPO*, *TSHR*, *TTF1*, *TTF2*, *PAX8*, *NKX2-5*, *GNAS*, *THRA*, *TSHB*, *IYD* and *SLC5A5*, to identify the spectrum and prevalence of disease-causing gene mutations in Chinese CH patients. CH-causing genes were detected by targeted next-generation sequencing in 106 CH infants. A total of 132 mutations were identified in 69 cases (65.1%). Of these 132 mutations, 92 (69.70%), 28 (21.21%), and 12 (9.09%) were related to thyroid dyshormonogenesis, thyroid dysgenesis, and thyrotropin resistance, respectively. Mutations in CH-causing genes were found mainly in *DUOX2*, *TG* and *TSHR*, and *DUOX2* is the most gene mutation in Chinese CH patients.

## Introduction

Congenital hypothyroidism (CH), which is defined by inadequate thyroid hormone production in newborn infants, is the most common neonatal metabolic disorder worldwide, with an incidence of 1 in 2000–4000 live births (Rastogi and LaFranchi, 2010). Most neonates born with CH have a normal appearance and no detectable physical signs. In the past, we have often overlooked the harmfulness of hypothyroidism during the newborn period. Patients with the disease suffer from delayed diagnosis and treatment, and severe CH can lead to growth retardation and permanent intellectual disability. CH screening is an important component of the newborn screening (NBS) program, which is widely used as the third prevention intervention of birth defects (Keskinkılıç,, 2014; Berry, 2015). Using the NBS program, we can detect symptomless children with CH early. Children can receive a definitive diagnosis, and the proper treatment can be applied in time to prevent further complications and sequelae. The NBS program was established in Changzhou city in 2004, and approximately 430,000 infants have since been tested.

Congenital hypothyroidism screening has been carried out all over the world for nearly 50 years, but the pathogenesis of CH remains unclear. An increasing amount of evidence suggests that genetic mutations are an important factor of CH (Nettore et al., 2013). At present, more than 600 genomic variations have been recorded in the ClinVar database. CH is divided into two main types: thyroid dysgenesis and thyroid dyshormonogenesis. According to previous reports, the cause of CH in approximately 80–85% of patients is thyroid dysgenesis (including agenesis, ectopy, and hypoplasia), which is related to gene mutations in thyroid-stimulating hormone receptor (*TSHR*), paired box gene 8 (*PAX8*), thyroid transcription factor 1 (*TTF1/NKX2-1*), thyroid transcription factor 2 (*TTF2/FOXE1*), and NK2 transcription factor related locus 5 (*NKX2-5*). Otherwise, 10–15% of cases are caused by thyroid dyshormonogenesis, which is associated with mutations in thyroid oxidase 2 (*DUOX2*), dual-oxidase maturation factor 2 (*DUOXA2*), thyroglobulin (*TG*), thyroid peroxidase (*TPO*), solute carrier family 5 member 5 (*SLC5A5*), solute carrier family 26 member 4 (*SLC26A4*) and iodotyrosine deiodinase (*IYD*) (Nettore et al., 2013; Cherella and Wassner, 2017). These genes play important roles in the growth and development of the thyroid gland. Genomic variations can prevent or destroy normal development of the gland or disturb the production of thyroid hormones. However, most previous studies have focused on Western populations. Few similar studies have been reported in a Chinese population and have focused on one or two pathogenicity genes (Fu et al., 2016b; Hu et al., 2016; Kizys et al., 2017). There are few reports on the mutation spectrum of CH-causing genes in the Chinese population.

In the present study, we retrospectively analyzed the clinical data of CH screening over the last 14 years and performed mutation screening of CH-causing genes in CH infants using next-generation sequencing (NGS). We hope to improve CH neonatal screening and better characterize the mutations of CH-causing genes in a Chinese population.

## Materials and Methods

### Patients and Design

From January 2004 to December 2016, all newborn infants who accepted the NBS program in Changzhou Maternity and Child Health Care Hospital Affiliated to Nanjing Medical University were recruited for this study. All subjects received CH screening via collection of dried blood spots (DBSs). In 2012, we began our search for CH-causing genes. One hundred and six non-consanguineous patients diagnosed with CH consented to undergoing the gene mutation test.

The study design and protocol were reviewed and approved by the ethics committee of Changzhou Maternity and Child Health Care Hospital Affiliated to Nanjing Medical University.

### NBS Program

The methods of screening, diagnosis, and treatment were carried out according to the “Subspecialty Group of Endocrinologic et al. (2011), which was promulgated by the Chinese Preventive Medicine Association. Briefly, DBS were collected from all infants on 903 filter paper (Wallac OY, Turku, Finland) at 72 h after birth. The levels of neonatal thyroid-stimulating hormone (NTSH) of DBS were detected by a time-resolved fluoroimmunoassay using the Wallac 1420 or Wallac 1235 AutoDELFIA (Perkin Elmer, Waltham, MA, United States). If the NTSH level was <9.0 mIU/L, the infants were considered normal. If the NTSH level was ≥9.0 and <20.0 mIU/L, the infants were recalled, and DBSs were collected for a second time (within 1 week) and the NTSH level re-determined. If the NTSH level was still >9.0 mIU/L, the infants were recalled again. The infant was considered normal if the second NTSH level was <9.0 mIU/L. If the NTSH level was ≥20.0 mIU/L, the infants were recalled, and the levels of serum thyroid-stimulating hormone (TSH) and free thyroxine (FT4) were determined.

### Diagnosis of CH

Venous blood from the recalled infants in the NBS program was sampled to test the concentrations of TSH and FT4. Diagnosis of CH was based on elevated TSH levels (TSH ≥ 10 mIU/L) and decreased FT4 levels (FT4 < 7.77 pmol/L). Serum TSH and FT4 levels were determined by electrochemistry immunoassay (ECL) using the COBAS e601 automated analyzer (Roche Diagnostics, Mannheim, Germany).

### Targeted Next-Generation Sequencing

Genomic DNA was extracted from peripheral blood leukocytes using the QIAamp DNA blood kit according to the manufacturer’s protocol. A total of 10 ng DNA per sample was used for sequencing using the CH capture panel, which was designed based on the Illumina Truseq Custom Amplicon v1.5 kit. Thirteen pathogenic genes (*DUOX2*, *TG*, *TPO*, *TSHR*, *TTF1*, *TTF2*, *PAX8*, *NKX2-5*, *GNAS*, *THRA*, *TSHB*, *IYD,* and *SLC5A5*) were screened in all patients, including the entire coding regions and exon-intron boundaries. The genetic fragments were between 250 and 280 bp and were prepared using the Covaris and Agencourt AMPure XP kits, which include purified and captured gene fragments. Adaptor-ligated amplicons were prepared using the Illumina Paired-End Sample Preparation kit. Illumina multi-PE-adaptors were bound to terminal genes and target enrichment was performed by multiplex PCR. After 12 PCR cycles, amplicons were purified using Agencourt AMpurc SPRI XP beads and captured on the Illumina MiSeq 2000 instrument.

### Mutation Analysis

Illumina Amplicon Viewer v1.3 and MiSeq Reporter v2.3 software were used for data analysis, and SnpEff was used for mutation annotation. We also used automatic tools (including SIFT, PolyPhen-2, and MutationTaster) to predict the impact of mutations on the function and structure of their respective proteins. Briefly, mutations with frequencies > 1% or synonymous mutations were filtered. The evaluation of potential pathogenic mutations was based on the combination of the three tools, and mutations for which 2 of the 3 tools predicted damaging effects were selected. We also searched the selected mutations in other published studies to evaluate their potential pathogenicity.

### Statistical Analysis

Data that were not normally distributed are expressed as medians (M), 25^th^ percentiles (P25), and 75^th^ percentiles (P75). All data were analyzed using EmpowerStats x64 software (Wu et al., 2015).

## Results

A total of 437,342 newborns, including 236,820 males and 200,522 females, underwent CH screening. In total, 3,931 infants had positive results, and their NTSH levels were in the range of 9.0–20.0 mIU/L. After being recalled, 3,768 infants underwent the second DBS test. Otherwise, 289 infants were considered as positive because their NTSH levels were >20.0 mIU/L. The positive rate of initial screening was 0.96% (4220/437342); 181 cases were missing in the first recall. The positive recall rate of initial screening was 95.7% (4039/4220). The NTSH levels of 501 recalled cases were still more than 9.0 mIU/L, and the positive rate of the recalled cases was 13.3% (501/3768). A total of 714 cases underwent the serological confirmation test, and 192 infants (105 males and 87 females) were diagnosed with CH. The incidence of CH was 1:2278 (1:2255 for males and 1:2304 for females). The positive predictive value was 4.8% (192/4039). A total of 239 infants were lost to follow-up. The loss to follow-up rate was 0.05% (239/437342). Reasons for loss to follow-up included incomplete contact information, dissent of the parents, or the patient moved to a new residence.

We examined the distribution of thyroid hormone levels (Table 1). The median NTSH level of the CH infants was 46.10 mIU/L (P25–P75: 17.90–120.00). The NTSH level of the healthy infants was 2.39 mIU/L (P25–P75: 1.37–3.93). The NTSH level of 99.04% of the normal infants was <9.0 mIU/L. The NTSH level was >100 mIU/L in 30.73% and 30 to 100 mIU/L in 28.13% of the CH infants. After serological examination, the median serum TSH level of CH infants was 75.0 mIU/L (P25–P75: 75.00–75.00), and that of FT4 was 5.14 pmol/L (P25–P75: 2.83–8.39). In total, 80.73% of CH cases had TSH levels >75 mIU/L.

**Table 1:** Distribution of thyroid hormone levels.

Range	*n*	%	Median	P25–P75
**NTSH in dried blood spots in all infants**				
Total	437342	100	2.39	1.37–3.93
<3	220272	61.80		
3–<6	132383	30.27		
6–<9	30467	6.97		
9–<12	3208	0.73		
≥12	1012	0.23		
**NTSH in dried blood spots in CH infants**				
Total	192	100	46.10	17.90–120.00
<9	5	2.60		
9–<15	35	18.23		
15–<50	59	30.73		
50–<100	34	17.71		
00–<200	42	21.88		
≥200	17	8.85		
**Serum TSH in CH infants**				
Total	192	100	75.00	75.00–75.00
10–<20	2	1.04		
20–<30	4	2.08		
30–<50	15	7.81		
50–<75	16	8.33		
≥75	155	80.73		
**Serum FT4 in CH infants**				
Total	192	100	5.14	2.83–8.39
<2.0	36	18.75		
2.0–<4.0	36	18.75		
4.0–<6.0	48	25.00		
6.0–<8.0	27	14.06		
8.0–<10.0	23	11.98		
10.0–<20	22	11.46		


Congenital hypothyroidism-causing genes were detected by targeted NGS in 106 CH infants. Based on our literature review, we designed a target sequencing panel, which included 13 causative genes: *DUOX2*, *TG*, *TPO*, *TSHR*, *TTF1*, *TTF2*, *PAX8*, *NKX2-5*, *GNAS*, *THRA*, *TSHB*, *IYD*, and *SLC5A5*. Among 106 CH infants, 69 (65.1%) had more than one gene mutation, and a total of 132 mutations were identified in nine genes (*DUOX2*, *TG*, *TPO*, *TSHR*, *TTF1*, *TTF2*, *NKX2-5*, *PAX8*, and *GNAS*). No mutation in *THRA*, *TSHB*, *IYD*, or *SLC5A5* was detected. In total, 92 of 132 (69.70%) mutations were related to thyroid dyshormonogenesis [*DUOX2* (*n* = 49), *TG* (*n* = 35), and *TPO* (*n* = 8)]. Additionally, 21.21% (28/132) of mutations were related to thyroid dysgenesis [*TSHR* (*n* = 19), *TTF1* (*n* = 5), *TTF2* (*n* = 1), *PAX8* (*n* = 2), and *NKX2-5* (*n* = 1)]. Moreover, 9.09% (12/132) of mutations were related to *GNAS*, which is associated with thyrotropin resistance (Mantovani et al., 2007). We made a list of the top 10 gene mutations detected in our study (Table 2). It is worth noting that the majority of mutations in the CH-causing genes were in *DUOX2*, *TG*, and *TSHR*, especially *DUOX2*. There were a total of 48 *DUOX2* mutations detected in our study, and 33 of 106 CH infants harbored *DUOX2* mutations. Twenty-four different types of *DUOX2* mutations were detected, including 20 reported mutations and four novel mutations (c.3721A > T, c.3321delC, c.1007_1009del, and c.1300_1320del). We also found 29 novel mutations, each with a rare frequency (Table 3).

**Table 2:** Top 10 of genes mutations in our study.

Gene_symbol	CytoBand	Exon position	Nucleotide position	Amino acid position	Mutation types	Number	RS ID
*TG*	8q24.22	Exon45	c.7847A > T	p.N2616I	Non-synonymous	11	rs10091530
*DUOX2*	15q21.1	Exon14	c.1588A > T	p.K530X	Stopgain	6	rs180671269
*DUOX2*	15q21.1	Exon30	c.4027C > T	p.L1343F	Non-synonymous	5	rs147945181
*DUOX2*	15q21.1	Exon25	c.3329G > A	p.R1110Q	Non-synonymous	5	rs368488511
*DUOX2*	15q21.1	Exon28	c.3632G > A	p.R1211H	Non-synonymous	4	rs141763307
*TG*	8q24.22	Exon42	c.7364G > A	p.R2455H	Non-synonymous	4	rs2272707
*DUOX2*	15q21.1	Exon4	c.227C > T	p.P76L	Non-synonymous	3	rs767705906
*DUOX2*	15q21.1	Exon26	c.3478_3480del	p.1160_1160del	Non-frameshift	3	rs758318135
*DUOX2*	15q21.1	Exon6	c.605_621del	p.Q202fs	Frameshift	3	rs769318570
*TSHR*	14q31.1	Exon10	c.1349G > A	p.R450H	Non-synonymous	3	rs189261858


NCBI Reference Sequence: TG (NM_003235), DUOX2 (NM_014080), TSHR (NM_000369).

**Table 3:** Novel mutations in our study.

Gene_symbol	CytoBand	Exon position	Nucleotide position	Amino acid position	Mutation types	Number
***TG* (NM_003235)**						
	8q24.22	Exon34	c.6185G > A	p.W2062X	Stopgain	2
	8q24.22	Exon8	c.976C > T	p.Q326X	Stopgain	1
	8q24.22	Exon8	c.1000delG	p.G334fs	Frameshift	1
	8q24.22	Exon10	c.2593C > A	p.P865T	Non-synonymous	1
	8q24.22	Exon16	c.3457A > T	p.K1153X	Stopgain	1
	8q24.22	Exon16	c.3538C > T	p.Q1180X	Stopgain	1
	8q24.22	Exon18	c.3994C > T	p.Q1332X	Stopgain	1
	8q24.22	Exon25	c.5020C > A	p.P1674T	Non-synonymous	1
	8q24.22	Exon45	c.7799G > A	p.W2600X	Stopgain	1
***DUOX2* (NM_014080)**						
	15q21.1	Exon9	c.1007_1009del	p.336_337del	Non-frameshift	1
	15q21.1	Exon12	c.1300_1320del	p.434_440del	Non-frameshift	1
	15q21.1	Exon25	c.3321delC	p.T1107fs	Frameshift	1
	15q21.1	Exon29	c.3721A > T	p.I1241F	Non-synonymous	1
***TSHR* (NM_000369)**						
	14q31.1	Exon1	c.152C > A	p.P51Q	Non-synonymous	1
	14q31.1	Exon6	c.501C > G	p.I167M	Non-synonymous	1
	14q31.1	Exon9	c.700T > C	p.S234P	Non-synonymous	1
	14q31.1	Exon10	c.1384T > C	p.C462R	Non-synonymous	1
***TTF1* (NM_007344)**						
	9q34.13	Exon2	c.269G > A	p.R90K	Non-synonymous	2
	9q34.13	Exon2	c.515A > G	p.Q172R	Non-synonymous	1
	9q34.13	Exon4	c.1598C > T	p.A533V	Non-synonymous	1
***GNAS* (NM_000516)**						
	20q13.32	Exon4	c.308T > C	p.I103T	Non-synonymous	2
	20q13.32	Exon6	c.478C > T	p.R160C	Non-synonymous	1
	20q13.32	Exon12	c.1018T > C	p.F340L	Non-synonymous	1
***PAX8* (NM_013953)**						
	2q13	Exon4	c.275T > C	p.I92T	Non-synonymous	1
	2q13	Exon5	c.398G > A	p.R133Q	Non-synonymous	1
***TPO* (NM_175722)**						
	2p25.3	Exon13	c.2080T > C	p.S694P	Non-synonymous	1
***NKX2-5* (NM_001166176)**						
	5q35.1	Exon2	c.416G > A	p.S139N	Non-synonymous	1


Among the 69 CH infants with mutations, 28 (40.58%) had one potentially functional mutation, 26 (37.68%) had two mutations, and 15 (21.74%) had three or more mutations. Similarly, 39 (56.52%) infants had mutations in one gene, and 30 (43.483%) had mutations in two or three genes.

## Discussion

The NBS program for CH is a major method used in preventive medicine. In this study, we retrospectively analyzed the clinical data from NBS over the last 14 years with the goal of improving CH neonatal screening. According to our results, the incidence of CH in Changzhou is 1:2278, which is the average level in China (Zhong et al., 2016). The National Centre for Clinical Laboratories reported an incidence of CH was 1:2281 based on the data of the 202 laboratories around China. Wassner and Brown (2015) reported that the apparent incidence of CH has more than doubled in recent years ranging from 1:2800 to 1:1400. Meanwhile, countries, such as the United States (Mitchell et al., 2011), Canada (Deladoëy et al., 2011), New Zealand (Heather et al., 2017), Scotland (Mansour et al., 2017), Brazil (Silvestrin et al., 2017), reported slight differences. In our study, there was no significant difference in the incidence of CH between males and females, consistent with the study by Zhao et al. (2016).

In the present study, we detected mutations of CH-causing genes in a Chinese population by targeted NGS and found that the abnormal rates of these related genes in Chinese CH patients was 65.1%. A total of 132 gene mutations were detected (69.70, 21.2, and 9.09% mutations were related to thyroid dyshormonogenesis, thyroid dysgenesis, and thyrotropin resistance, respectively). This result was quite different from previous reports. According to research in Western countries, the primary pathology of CH is thyroid dyshormonogenesis (Nettore et al., 2013; Cherella and Wassner, 2017). The pathogenic factors of CH in our Chinese population may differ from those in Western populations. It is very important to screen the pathogenic genes and pathogenic factors of CH in this region. In addition, the current study indicated that a considerable proportion of Chinese CH patients had mutations at multiple sites or in multiple genes. Multiple mutations may cause a more serious phenotype in CH patients. Recent studies have also revealed that a significant proportion of CH patients have multiple gene variations in more than one thyroid-specific gene (de Filippis et al., 2017). Moreover, heritable variations were found in more than half of our CH patients, as well as in the general population, albeit at a significantly lower prevalence. Together, these studies indicate that the pathogenesis of CH may be due to the sum effect of rare alleles (Persani et al., 2018). A previous study also indicated that patients with one or two *DUOX2* pathogenic mutations developed subclinical or transient CH, whereas patients with three or more *DUOX2* pathogenic mutations were associated with permanent CH (Matsuo et al., 2016). The coexistence of multiple pathogenic mutations may contribute to the severity of the hypothyroid condition, and mutations in multiple genes may lead to genotype–phenotype variability (Moreno et al., 2002; O’Neill et al., 2015; Zheng et al., 2016). Therefore, further studies are needed to enlarge the mutation spectrum of CH and to verify the functions of the associated mutations, which may provide more profound insight into the etiology of CH.

In the present study, *DUOX2* was the most commonly mutated gene in Chinese CH infants. According to previous studies, mutations in *DUOX2* are responsible for thyroid dyshormonogenesis (Moreno and Visser, 2007). Most patients with *DUOX2* pathogenic mutations have an ectopic thyroid gland with an increased or normal size (Kizys et al., 2017). However, the mutational spectrum of the *DUOX2* gene and the correlations between phenotype and genotype have not yet been fully established. The c.1588A > T mutation in *DUOX2*, which is responsible for thyroid dyshormonogenesis, was highly recurrent, with a prevalence of 1/40,000. The c.1588A > T mutation is population specific and has been reported mainly in Asian populations, including Chinese (Fu et al., 2015, 2016a; Tan et al., 2016), Japanese (Maruo et al., 2008, 2016), and Malaysian (Chow et al., 2017) populations. The c.4027C > T (Chen et al., 2018), c.3329G > A (Fu et al., 2015; Park et al., 2016), c.3632G > A (Chai et al., 2015), c.2335G > A (Jiang et al., 2016; Maruo et al., 2016), and c.2654G > A (Zheng et al., 2016) mutations are also predominant in Asians, mostly in the Chinese Han population c.1883delA (Maruo et al., 2008, 2016; Park et al., 2016; Tan et al., 2016), c.3478_3480del (Narumi et al., 2011; Fu et al., 2016a; Park et al., 2016), and c.605_621del (Jin et al., 2014; Matsuo et al., 2016; Tan et al., 2016) show a scattered distribution in Asian populations, including China, Japan, and South Korea. Six other mutations, including c.2048G > T (Fu et al., 2016a), c.227C > T (Lv et al., 2011), c.2894C > T (Jiang et al., 2016), c.3391G > T (Wang et al., 2014; Fu et al., 2016a), c.2202G > A (Wang et al., 2014) and c.2104_2106del (Fu et al., 2016a), were reported only in China, and the missense mutation of c.4405G > A, which was previous reported in Korean (Park et al., 2016), was first identified among Chinese population in our study. The c.1873C > T mutation was identified as a novel pathogenic mutation by the qCarrier test in a reproductive carrier-testing program (Abulí et al., 2016), but no direct evidence has shown that the mutation is related to CH. In addition, c.1265G > A, c.2413G > A, c.1717C > T, c.3721A > T, c.3321delC, c.1300_1320del, and c.1007_1009del mutations, which may be related to CH, were identified in our study for the first time.

## Conclusion

The incidence of CH in Changzhou city is 1:2278. Some related quality control indicators indicate that the NBS program of CH in Changzhou is effective. Meanwhile, we preliminarily identified the pathogenic genes in infants with CH by targeted NGS. The rate of abnormal gene mutations was 65.1%, and most mutations were related to thyroid dyshormonogenesis, which differs from that observed in Western populations. A considerable proportion of the population had mutations at multiple sites, and *DUOX2* was the most common gene mutation in Chinese CH infants.

## Author Contributions

BY, HW, and WL carried out the assays and participated in designing the study. HW and YW carried out clinical consultations. BY, YY, WL, and LJ carried out laboratory tests and performed the statistical analysis. ZC conceived the study, participated in its design and coordination, and helped draft the manuscript.

## Conflict of Interest Statement

The authors declare that the research was conducted in the absence of any commercial or financial relationships that could be construed as a potential conflict of interest.
